# Adolescents’ School Burnout: A Comparative Study between Italy and Switzerland

**DOI:** 10.3390/ejihpe11030062

**Published:** 2021-08-11

**Authors:** Piera Gabola, Nicolas Meylan, Marine Hascoët, Simona De Stasio, Caterina Fiorilli

**Affiliations:** 1 Unit of Teaching and Research, Development from Childhood to Adulthood, University of Teacher Education of Lausanne, 1014 Lausanne, Switzerland; nicolas.meylan@hepl.ch (N.M.); marine.hascoet@hepl.ch (M.H.); 2 Department of Human Studies-Communication, Education and Psychology, LUMSA University, 00193 Roma, Italy; s.destasio@lumsa.it (S.D.S.); fiorilli@lumsa.it (C.F.)

**Keywords:** burnout, adolescents, gender, Switzerland, Italy

## Abstract

This study aimed to analyze and compare students’ school burnout levels in Switzerland and Italy. Previous research has confirmed that female and older students in particular are highly exposed to burnout risk. Nevertheless, few studies have observed this phenomenon through a cross-national comparison. Data on burnout were collected from a sample of 840 adolescents (Italian students = 497; Swiss students = 343) (M*age* = 14.98; SD = 1.06; Female = 50%). Burnout was measured using the School Burnout Inventory, and cross-cultural measurement invariance was tested. The results showed that this burnout measure was equivalent between the Italian and Swiss samples. A multivariate analysis of variance was next conducted to investigate the effects of age, gender, and nationality. Results partially confirmed our hypotheses, showing the effect of age but not of gender in explaining burnout differences among students, and between and within-group variance. In particular, the burnout risk was found to be higher in late adolescence (age 16 to 18, M*_exhaution_* = 2.73; M*_cynicism_* = 2.99; M*_Inadequacy_* = 3.14) than in mid-adolescence (age 13 to 15 M*_exhaution_* = 2.95; M*_cynicism_* = 3.43; M*_Inadequacy_* = 3.54). Furthermore, Italian adolescents were more exhausted and cynical (M*_exhaution_* = 2.99; M*_cynicism_* = 3.26) than their Swiss peers (M*_exhaution_* = 2.52; M*_cynicism_* = 2.93) when controlling for age and gender. Findings suggest further investigation of the role played by educational and cultural values may be warranted.

## 1. Introduction

Students’ school burnout is a comprehensive scientific framework to interpret students’ attitudes toward their school life affecting their achievement and later academic career. Scholars agree with the well-known model describing students’ burnout through three dimensions: emotional exhaustion, cynicism, and sense of inadequacy [1,2]. Exhaustion is characterized by emotional fatigue and is generally due to highly demanding school tasks. The cynicism dimension represents the negative attitude toward school-life events and poor relationships with teachers and classmates. Finally, sense of inadequacy refers to how students can successfully cope with school challenges. Overall, school burnout arises when students feel overwhelmed and unable to cope with stressful events. When students feel exhausted, and without support to face school-related tasks, they may express maladaptive behaviors (e.g., absenteeism, bad grades, aggressive behaviors), with severe consequences for their future health [3,4,5,6]. More specifically, school burnout may affect upper-secondary students who face high academic expectations, on one hand, and perceived parental pressure, on the other. The imbalance between demand (e.g., by family and school) and internal and external resources (e.g., psychological characteristics and social support) may lead to a high burnout risk [7,8,9]. Conversely, personal resources, such as empathy, self-efficacy, resilience, and emotional intelligence, may significantly reduce burnout risk [10,11,12,13]. 

Students’ maladjustment to school life has been widely investigated in recent decades in different countries. For example, about 6.7% of Slovenian adolescents [4] show worrying burnout scores, as do between 10% and 15% of Finnish adolescents [14]. Up to 21% of Swiss adolescents display severe burnout levels [5], and more than 40% of French adolescents exhibit high degrees of burnout [6]. Concerning the Italian context, the rate of high burnout levels amounts to about 20% [7].

Interestingly, findings open up questions regarding what kind of contextual and educational conditions may contribute to making a difference among students around the world. In the case of young students, the social support they receive from others may enhance their personal resources (such as emotional intelligence and resilience), reducing burnout risk [15,16,17]. Previous studies have effectively demonstrated that positive relationships with parents, teachers, friends, and classmates are associated with a low degree of burnout [18]. Comparing different school systems in terms of the support offered to students’ wellbeing could be helpful to understand burnout degree differences around the world. In our opinion, it would be interesting for policymakers, teachers, and practitioners to deeply understand this phenomenon, which has been demonstrated to dramatically impact students’ academic careers. Furthermore, comparative research in this field may offer findings, practices, and tools to prevent and reduce students’ maladaptive behaviors at school. 

Interestingly, Italy and Switzerland share similar cultural values, but with different school system programs for their students’ wellbeing. 

Concerning cultural values, Hofstede’s criteria [19] highlighted that Italy and Switzerland display similar sociocultural values. Both countries share identical democracy and equality values, and their welfare systems ensure assistance and supportive aids for their citizens. More specifically, their values depend on their belonging to the same cultural sphere characterized by individualism versus collectivism. In this regard, the school system is the principal promoter and guide of countries’ cultural values (e.g., supporting competitive methods and promoting individual success).

Contrariwise, the core difference between the two countries refers to the school programs and interventions promoting students’ wellbeing. Even though there are some differences among Swiss cantons, every cantonal educational department offers a health team supporting each school. The main goal is to promote students’ health and wellbeing. Furthermore, they work to maintain positive relationships among student groups and between teachers and their classrooms. Some health professionals are, for example, the school nurse, school mediator, school doctor, and school psychologist. Overall, the health team may guarantee a quick and easy answer to students’ maladaptive behaviors by contributing to developing a wellbeing culture and offering a helping service devoted to each student.

By contrast, there are no stable health professionals inside the Italian school system. Apart from some specific school districts, the Italian school system does not offer psychosocial and medical support to their students. As is well known in Italy, there is a very long tradition of inclusion of students with special needs taught by specialized teachers who work with the regular teacher and the whole classroom. Nevertheless, students with intimate and personal needs due to their private and school life can only rely on sensitive and responsive teachers.

To understand students’ differences in school burnout experience, age and gender have received extensive attention. Nevertheless, findings have been inconsistent, which could be due to the lack of comparative studies conducted with the same instruments of measure. Concerning students’ age, previous results lead us to consider the double role of the growing up effect, on one hand, and the school transition effect, on the other. For example, a study conducted in Korea with 1530 young students showed that burnout levels rise gradually with age.

Consequently, transitioning from elementary to middle, then to high school becomes increasingly stressful [20]. School burnout increases among Finnish students during the transition from comprehensive school to senior high school, especially cynicism and inadequacy [14]. Very high levels of inadequacy have been recorded in a Swiss study carried out with 380 adolescents (aged 14–19) at the beginning of post-compulsory schooling [5]. Similarly, more senior students were found to be more at risk of burnout in a recent Italian context study than younger ones [13]. Overall, previous comparative and international findings have abundantly confirmed that the more students grow up, the more demanding school requests they face [21].

Regarding the relationship between school burnout and gender, prior research has pointed out gender differences in emotional engagement with childhood schooling. Several studies have highlighted that girls and boys are exposed to school burnout risk in different ways [5,14,22,23]. More specifically, boys show more behavioral problems and externalized symptoms, such as cynicism [24], whereas girls experience more internalized symptoms, such as inadequacy and exhaustion e.g., [25]. Being male is a predictor for being more disengaged at the start of secondary school and throughout it. The school disengagement of male students has been attributed to many aspects, including boys’ desire to gain popularity through dismissing schoolwork and their aversion to subjects that lack a practical component [26]. School burnout in girls is frequently the outcome of high levels of engagement and outstanding academic success that may come at the price of exhaustion and diminished overall wellbeing [27].

This result agrees with findings that have substantially supported partially overlapping burnout and depression symptoms. Women are more likely affected by depression than men [11,28]. Interestingly, findings suggest that even though girls are more burned out than boys, they also show better achievement and attribute greater importance to academic achievement than boys (e.g., [5,26,29,30]). On the other hand, some other findings (e.g., [31]) support no consistent association between students’ gender and burnout level, calling for further research on the link between burnout and gender. For example, the study in [2] showed high cynicism among the female group, which other scholars did not find [26,32].

### Aims and Hypotheses

The current study’s central core was examining school burnout differences between Swiss and Italian adolescents (i.e., exhaustion, cynicism, and inadequacy), considering their gender and age variables. The target population was adolescents between 12 and 18 years old and attending Swiss and Italian public schools. 

The above literature has shown the critical role played by students’ background variables to explain students’ school burnout differences beyond culture dimensions (i.e., being female and older exposed students to a higher level of school burnout). In this regard, we expected the same role to be played by gender and age variables in the Italian and Swiss student samples. Firstly, we expected female and male students to show significant differences in the three school burnout dimensions. More specifically, females were expected to lead, overall, with higher burnout scores than their male colleagues. Secondly, we expected that older students would show higher school burnout levels than younger students. Finally, since Swiss schools provide a permanent in-school service for promoting and maintaining students’ health, something which is absent in Italy, we were interested in exploring the differences between Italian and Swiss students’ overall wellbeing. 

## 2. Methods

### 2.1. Participants and Procedure 

Initially, 875 adolescents responded to a questionnaire with several scales regarding school and health. A search for outliers (e.g., same response to all items) indicated a need to delete 17 subjects. Moreover, participants with missing values corresponding to more than 5% of the items were excluded (listwise deletion method) and represented 2.1% of the participants. 

The final sample was composed of 840 adolescents ranging in age from 13 to 18 (M = 14.98; SD = 1.06), recruited from high schools in comparable urban settings in each of the two countries to maximize the homogeneity of the social–demographic variables. The Italian sample was composed of 497 adolescents (male = 257; female = 240; mean age M = 15.09; SD = 0.99) from two urban schools. The Swiss sample was composed of 343 adolescents from two urban schools (male = 174; female = 169; mean age M = 14.81; SD = 1.12). Migrant students and students with disabilities and/or learning disabilities were excluded from the sample for this study. Parents’ written consent and information about eligibility for participation were obtained for those who agreed to participate. Trained researchers administered the measures during school hours. The school council approved the study before the administrations. All the study procedures followed the Declaration of Helsinki of 1964 and its latest version. The Ethics Committee approved the research protocol shared by Lumsa University (Rome, Italy) and the University of Teacher Education Vaud (Lausanne, Switzerland). The research was conducted following the APA ethical principles and code of conduct. All data were collected during the academic year of 2020. 

### 2.2. Instruments

The School Burnout Inventory is a questionnaire developed and validated in both Italian and French [7,33]. It consists of nine items measuring three components of school burnout: (1) exhaustion at school (e.g., “I feel overwhelmed by my schoolwork”); (2) cynicism toward the meaning of school (e.g., “I am not motivated to do my schoolwork and often think of giving up”), and (3) sense of inadequacy at school (e.g., “I usually have feelings of inadequacy about my schoolwork”), to be rated on a 6-point scale (1 = strongly disagree; 6 = strongly agree). Two self-report items concerning age and gender were also administered to all students.

### 2.3. Strategy of Analysis

Before burnout latent means can be compared between the two samples, it is necessary to ensure that the burnout scale is equivalent for both populations. In other words, it must be confirmed that the same concept is measured for Swiss and Italian students. With this objective, preliminary analyses were conducted to evaluate the cross-cultural measurement invariance of the School Burnout Inventory. Next, the distribution of burnout scores was studied. Furthermore, dummy variables were obtained for age (mid-adolescence: 13 to 15 years old; late adolescence: 16 to 18 years old) and gender. Finally, a series of analyses of variance (ANOVAs) and multivariate analyses of variance (MANOVAs) were performed to assess differences related to gender and age in adolescents’ school burnout in both countries. Analyses were performed using the *Lavaan package* for R software [34] and SPSS statistics 21. 

## 3. Results

### 3.1. Cross-Cultural Measurement Invariance 

To test the equivalence of the burnout measure between Swiss and Italian samples, we first examined separate measurement models for Swiss students (Model A1) and Italian students (Model 1B). We used indices to evaluate the fit of our model [35,36,37]. We first reported Chi-squares and their significance. To confirm the good fit of our data, Chi^2^ must be nonsignificant. However, as is often the case with large samples, some authors conclude that this index does not provide a practical model fit test [38,39]. We also considered the standardized root mean square residual (SRMR), the root mean square error of approximation (RMSEA), and the comparative fit indices (CFI). We also considered McDonald’s noncentrality index (NCI). The CFI and the NCI allow testing cross-group differences in multigroup confirmatory factor analysis [39]. The values of SRMR and RMSEA are good up to 0.08. The NCI and CFI are acceptable if they are superior to 0.90 and excellent if they are superior to 0.95. 

We then tested the cross-cultural measurement invariance of scales and performed multigroup confirmatory factor analysis [40]. To do so, three nested models with increasingly restrictive assumptions needed to be tested. The configural invariance (model C) tests the identical configuration of parameters across groups and provides the basis for comparison with the subsequent model [40,41]. The metric invariance model (model D) constrained the factor loadings to be equal across groups. This model allows testing if the relationship between indicators and latent scores is the same between the Swiss and the Italian samples. The last model (model E) is the scalar invariance model. In this model, intercepted items are also constrained to be the same across groups. However, the full measurement invariance frequently does not hold, and partial scalar invariance (for example, if an item has an invariant loading but a different cross-group intercept) might be sufficient to test the comparison factor mean [40]. Because of the increase in constraints, the model fit will decrease [42]. The assumption of invariance if the differences between the indices from the base model and the ones from the more constrained model fall within the following thresholds. Because of the sample sensitivity of the Chi^2^ test, [40] recommend accepting a more constrained model if the CFI difference is less than 0.01 and the NCI difference is less than 0.02. The authors of [43] suggest that the change in the RMSEA should not be greater than 0.015 and that the change in the SRMR should not be greater than 0.030 for testing metric invariance and 0.10 for testing scalar invariance. For each model, we reported the values of the fit indices (Table 1) and the differences between indices from this model and the less constrained ones (Table 2). Models A1 and A2 showed that measurement models had a good fit for the two groups even if the fit indices are slightly better for the Italian sample. Model C tests the configural invariance. This model showed a good general fit: the CFI and the NCI indices are good (0.947 and 0.946, respectively), including the RMSEA and the SRMR (0.068 and 0.044, respectively). Configural invariance could be established, and the structure of the measure was found to be equal across the group. In model D, item loadings were constrained to be equal between groups to test metric invariance. The fit indices of this model were acceptable (χ^2^ = 162.828, df = 56, *p* < 0.001, CFI = 0.938, NCI = 0.938, RMSEA = 0.067, 90% CI[0.055; 0.080], SRMR = 0.059). A comparison between models D and C is presented in Table 2. The Chi^2^ difference test was significant, which means that model D had a worse adjustment than model C. However, this test is sensitive to sample size, and the ΔCFI was lower than recommended (0.009 < 0.10), which was also the case for the ΔRMSEA (0.001 < 0.015), the ΔSRMR (0.008< 0.030), and the ΔNCI (0.008 < 0.02). Therefore, we could assume the full metric invariance of our measure.

Model E1 tested full scalar invariance by constraining the intercepts of indicators to be equal between the Swiss and the Italian samples. This model showed a poor adjustment (χ^2^ = 240.713, df = 61, *p* < 0.001, CFI = 0.896, NCI = 0.898, RMSEA = 0.084, 90% CI[0.073; 0.095], SRMR = 0.069). Moreover, the comparison between this model and the previous one (Model D) showed that the ΔCFI is higher than the recommended value (0.042 < 0.10), which was also the case for the ΔRMSEA (0.017 < 0.015) and the ΔNCI (0.040 < 0.02). Only the ΔSRMR was at an acceptable level (0.008 < 0.010). Therefore, we could not confirm the full metric invariance of our measure.

We looked at the modification indices to see if some items’ intercepts were not invariant across the groups and then tested the partial scalar invariance model [36]. The modification indices showed that item 3, “I often have feelings of inadequacy in my schoolwork”, was not invariant. Consequently, we allowed the item 3 intercept in the model E2 to be unconstrained (i.e., to vary across groups). This model presented a better adjustment (χ^2^ = 198.416, df = 60, *p* < 0.001, CFI = 0.925, NCI = 0.926, RMSEA = 0.072, 90% CI[0.060; 0.083], SRMR = 0.063). Moreover, this model did not show a worse fit than model D, which tested the full metric variance: ΔCFI was very close to the recommended value (0.013 for 0.010), while the ΔRMSEA was within the recommended value (0.005 < 0.15), same as the ΔSRMR (0.003 < 0.010) and the ΔNCI (0.003 < 0.02).

Therefore, we can conclude a configural, full metric, and partial scalar invariance of our measure. All the factor loadings and all but one intercept are invariant between our two groups. The latent means are based on cross-nationally comparable items, and their variations across the two groups can be tested [41].

### 3.2. Descriptive Statistics for School Burnout Dimensions

Table 3 reports the central descriptive values of school burnout based on nationality (Swiss, Italian). Firstly, distributions of the school burnout scores show that all skewness and kurtosis coefficients are below the ± 1 threshold recommended by [44]. Secondly, the nature of school burnout was similar in the two subsamples for the three dimensions of school burnout (in order: inadequacy: Switzerland, M = 3.22; Italy, M = 3.29; cynicism: Switzerland, M = 2.93—Italy, M = 3.26, and exhaustion: Switzerland, M = 2.52—Italy, M = 2.99). Thirdly, concerning burnout levels, Italian adolescents (27.6%) were found to have higher burnout levels than their Swiss counterparts (14.6%).

### 3.3. Gender, Age, and Country Differences in School Burnout

Table 4 shows gender, age (13–15 years old, 16–18 years old), and country (Switzerland, Italy) differences in the School Burnout Inventory total score by computing a 2 (gender) χ^2^ (age) χ^2^ (nationality) ANOVA.

There was a main effect for age (*F*_(1, 840)_ = 21.46, *p* < 0.001) and nationality (*F*_(1, 840)_ = 17.07, *p* < 0.001) but not for gender, and no interaction was found between those variables. In other words, it appears that the school burnout total score is higher among Italian students than among Swiss students (*p* < 0.001) and among late adolescents than among mid-adolescents (*p* < 0.001). To investigate gender, age, and nationality effects on the school burnout dimensions, a 2 (gender) χ^2^ (age) χ^2^ (nationality) MANOVA was conducted on the School Burnout Inventory subscales (exhaustion, cynicism, inadequacy). Correlations between those subscales ranged from 0.33 to 0.59 (see Table 2). There was a main effect for age (Wilk’s λ = 0.974, *F*_(3, 830)_ = 7.46, *p* < 0.001) and nationality (Wilk’s λ = 0.958, *F*_(3, 830)_ = 12.06, *p* < 0.001) but not for gender, and no interaction effect. The results of follow-up ANOVAs for the age main effect indicated that late adolescents obtained higher exhaustion (*F*_(1, 840)_ = 8.32, *p* < 0.01), cynicism (*F*_(1, 840)_ = 16.98, *p* < 0.001), and inadequacy (*F*_(1, 840)_ = 16.21, *p* < 0.001) scores than mid-adolescents. Follow-up ANOVAs on the main effect for nationality indicated that Italian students have higher exhaustion (*F*_(1, 840)_ = 25.15, *p* < 0.001) and cynicism (*F*_(1, 840)_ = 10.04, *p* < 0.01) scores than Swiss students. Table 3 presents the results of ANOVA and MANOVA.

## 4. Discussion

The current study addressed school burnout differences in Swiss and Italian adolescent groups. Findings showed school burnout differences overall due to students’ age, while no differences were found between male and female students. More specifically, whereas overall levels of school burnout seem identical in Switzerland and Italy, the intensity of exhaustion and cynicism varied from one country to the other. Compared to their Swiss counterparts, Italian students showed higher fatigue levels and cynicism toward their school life. By contrast, findings suggest no differences in students’ sense of inadequacy. Overall, more senior students are at risk of experiencing school maladjustment in their school careers. Burnout risk, and its dramatic consequences, are higher for Italian students, both female and male.

To summarize, our findings partially confirmed the study’s hypothesis. Concerning the expected differences between girls and boys, the current research supports the inconsistent results concerning the gender effect.

Among the several reasons accounting for no gender effect in both countries, an exciting perspective comes from [45], which suggested that gender may play a moderating role between work conditions (assimilable to school-related dimensions) and burnout. In other words, it is not the fact of being female or male, which makes a difference per se, but how boys and girls interact with the social resources available to them (e.g., teachers, practitioners) [46,47].

For further research, it would be interesting to compare schools that offer social support to students with schools that do not and examine how this impacts students’ perception of being supported.

Regarding the interconnection between student burnout and the perception of social support, the results of a recent meta-analysis [8] highlighted that the relationship between overall student burnout and total social support was significant. Students who feel more burnout regarding their studies are likely to think that they are less supported by their significant others. Social support provides a buffering effect against stress in that an individual who perceives more social support is also more resilient to stress. In this regard, the research above has demonstrated the role of teachers’ sensitivity [10] and emotional intelligence [15]. Teachers’ emotional competencies play an essential role in two ways. First, teachers with a good emotion regulation process toward their worker-related events are more likely to experience positive emotions and low burnout levels. Second, teachers with positive attitudes toward their schoolwork become themselves valuable support for their students, reducing those students’ risk of maladaptive behavior.

Concerning age variables, our results show that older students (aged 17–19) are more at risk of school burnout than younger ones (aged 13–15). Researchers have outlined that as students move further along in their educational trajectories in school, burnout symptoms increase. Previous studies have shown that school burnout levels were higher after transitioning from elementary to high school [5,13]. This result seems to confirm earlier findings (e.g., [13]), according to which students become more at risk of both burnout and depressive symptoms as they grow older. Effectively, school tasks become more challenging for students from one year to the next in their academic careers.

Regarding the last hypothesis, our findings show that Italian students display a higher rate of SBI. In our opinion, the central role of health services devoted to students in the Swiss school system may account for this difference between the two countries. Future studies with more measurement points are needed to better understand the differences between the school burnout levels reported by Italian and Swiss students. It would be relevant, in future, to examine what makes Italian students more exhausted than their Swiss peers.

## 5. Conclusions

Two relevant results come from the current study, opening to some reflections. Firstly, contextual social factors (e.g., social support) should be analyzed more in depth in comparative studies examining adolescents’ wellbeing in school. Secondly, the current study offers suggestions to promote wellbeing among adolescents in school. We believe that school-based programs promoting positive education may reduce school burnout risk in adolescents [48]. General support for preventing school burnout should be offered to all students since, for example, supportive educational practices [49] and motivating teachers [1] are linked to lower school burnout symptoms among students. It seems advisable to educate parents and teachers on the fact that pushing too hard and placing excessive expectations on students may be just as likely to result in problems with wellbeing as it is to foster achievement in the long run. Furthermore, implementing practices that promote social–emotional learning in schooling may also contribute to students’ wellbeing [50].

## 6. Limitations

To overcome the current study’s limitations, further research should consider how cultural bias has contributed to differences in scores between groups, including cultural differences in item appreciation. Furthermore, a multimeasure and multi-informant approach will be more informative when it comes to students’ burnout risk. Finally, it will be of interest to analyze the same variables in a larger sample of students in the future and to compare more cultural contexts.

## Figures and Tables

**Table 1:** Fit indices from the comparative factor analysis (CFA) and invariance analyses between groups.

	χ^2^(df)	CFI	NCI	RMSEA [90% CI]	SRMR
*Single group CFA*					
Model A1: Swiss students	86.478 (24)	0.927	0.913	0.087 [0.068; 0.107]	0.059
Model A2: Italian students	53.948 (24)	0.966	0.970	0.05 [0.032; 0.068]	0.04
*Multiple group CFA*					
Model C: Configural invariance	140.42 (48)	0.947	0.946	0.068 [0.055; 0.081]	0.044
Model D: Full metric invariance	162.828 (56)	0.938	0.938	0.067 [0.055; 0.080]	0.059
Model E1: Full metric and full scalar invariance	240.713 (61)	0.896	0.898	0.084 [0.073; 0.095]	0.068
Model E2: Full metric and partial scalar invariance (item 3 free)	198.416 (60)	0.925	0.926	0.072 [0.060; 0.083]	0.062

Note. All the χ^2^ tests are significant (*p* < 0.001).

**Table 2:** Fit index of the comparison between the models of invariance.

	Δ χ^2^( (df)	ΔCFI	ΔRMSEA	ΔSRMR	ΔNCI
Model D (Full metric invariance) vs. Model C (Configural invariance)	22.41 (8)	−0.009	−0.001	0.015	−0.008
Model E1 (Full metric and full scalar invariance)*vs.* Model D (Full metric invariance)	77.88 (5)	−0.042	0.017	0.009	−0.040
Model E2 (Full metric and partial scalar invariance) vs. Model D (Full metric invariance)	26.58 (4)	−0.013	0.005	0.003	−0.003

Note. All the Δ χ^2^ are significant (*p* < 0.001).

**Table 3:** Descriptive statistics, reliability, and rate of level of school burnout.

Variable		Mean	SD	Alpha	Skewness	Kurtosis	High Level of Burnout
Italy	Total score	28.34	8.51	0.74	0.02	−0.25	27.6% (N = 137)
	Exhaustion	2.99	1.06	0.55	0.25	−0.31	
	Cynicism	3.26	1.42	0.76	0.15	−0.93	
	Inadequacy	3.29	1.33	0.75	0.00	−0.75	
Switzerland	Total score	25.30	8.20	0.82	0.33	−0.31	14.6% (N = 50)
	Exhaustion	2.52	0.96	0.66	0.66	0.18	
	Cynicism	2.93	1.24	0.77	0.35	−0.64	
	Inadequacy	3.22	1.19	0.57	0.17	−0.57	
Overall	Total score	27.10	8.51	0.77	0.15	−0.35	22.3% (N = 187)
	Exhaustion	2.80	1.05	0.61	0.42	−0.25	
	Cynicism	3.13	1.36	0.76	0.25	−0.82	
	Inadequacy	3.26	1.27	0.76	0.06	−0.68	

*Note.* err std skewness IT = 0.110, CH = 0.132, overall = 0.084; err std kurtosis IT = 0.219, CH = 0.263, overall = 0.169; Italy N = 497; CH = 343, overall N = 840.

**Table 4:** School burnout in Italy and Switzerland.

Sample	Total		ANOVA Results	Exhaustion		Cynicism		Inadequacy					
	M	SD		M	SD	M	SD	M	SD	Main Effect	Interaction Effect	Follow up ANOVA	Comparisons
Gender			*F*_(1, 840)_ = 0.54, n.s.							Wilk’s λ = 0.996, *F*_(3, 830)_ = 1.14, n.s	n.s.		
Boys	27.33	8.36		2.82	1.03	3.14	1.37	3.3	1.26				
Girls	26.77	8.72		2.76	1.07	3.11	1.35	3.21	1.28				
Age			*F*_(1, 840)_ = 21.46, *p* < 0.001							Wilk’s λ = 0.974, *F*_(3, 830)_ = 7.46, *p* < 0.001, partial eta squared = 0.03		EXH: F (1, 840) = 8.32, *p* < 0.01	late ado > mid ado
13–15	26.17	8.55	late ado > mid ado	2.73	1.04	2.99	1.33	3.14	1.29			CYN: F(1, 840) = 16.98, *p* < 0.001	
16–18	29.18	8.08		2.95	1.05	3.43	1.38	3.54	1.18			INAD: F(1, 840) = 16.21, *p* < 0.001	
Nationality			*F*_(1, 840)_ = 17.07, *p* < 0.001							Wilk’s λ = 0.958, *F*_(3, 830)_ = 12.06, *p* < 0.001, partial eta squared = 0.04		EXH: F (1, 840) = 25.15, *p* < 0.001	It > CH
Italian	28.34	8.51	IT > CH	2.99	1.06	3.26	1.42	3.29	1.33			CYN: F (1, 840) = 10.04, *p* < 0.01	
Swiss	25.3	8.20		2.52	0.96	2.93	1.24	3.22	1.19				

## Data Availability

Not applicable.
